# Efficacy of interleukin-1 blockade in Schnitzler’s syndrome without detectable monoclonal gammopathy: a case-based review

**DOI:** 10.1007/s10067-020-05501-w

**Published:** 2020-11-11

**Authors:** Riccardo Bixio, Maurizio Rossini, Alessandro Giollo

**Affiliations:** Rheumatology Section, Department of Medicine, University of Verona, Policlinico G.B. Rossi, Piazzale L.A. Scuro 10, 37134 Verona, Italy

**Keywords:** Anakinra, Canakinumab, IL-1 blockade, Monoclonal gammopathy, Paraprotein, Schnitzler’s syndrome

## Abstract

Schnitzler’s syndrome (SchS) is a rare autoinflammatory disorder characterized by urticarial rash and monoclonal gammopathy which is currently regarded as IL-1 mediated disease. We present the case of a 21-year-old woman presenting with urticarial rash, arthralgias, and elevated inflammatory markers. She has been suffering these symptoms for 2 years and was treated with antihistamines, omalizumab, steroids, and non-steroidal anti-inflammatory drugs (NSAIDs) without success. After an extensive diagnostic workout, we suspected SchS even without monoclonal gammopathy, and started Anakinra 100 mg daily with a dramatic response and achieving complete remission after 48 h of the beginning of the treatment, so we decided to confirm SchS diagnosis. We performed a search of the literature and found seven more cases of patients diagnosed with SchS without monoclonal gammopathy at the presentation. Five were treated with IL-1 blocking therapies and all achieved remission. We, therefore, prompt the possible role of IL-1 blockade therapy remission as support in diagnosing SchS without monoclonal gammopathy.

## Introduction

Schnitzler’s syndrome (SchS) is a rare autoinflammatory disease described by L. Schnitzler in 1972 [1] characterized by urticarial rash and monoclonal gammopathy, usually Immunoglobulin (Ig) M or, rarely, IgG, associated with fever, joints and bones pain, enlarged lymph nodes, leukocytosis, and systemic inflammation (usually erythrosedimentation rate (ESR) and C-reactive protein (CRP) elevation) [2]. Long-term complication includes the development of lymphoproliferative diseases and amyloidosis. Two different diagnostic criteria (Lipsker’s and Strasbourg) have been developed and validated in real-life patients both with high sensitivity and specificity [3].

SchS diagnosis is challenging due to the rarity of the disease, and diagnostic workout usually needs to rule out many inflammatories, allergic, genetic, infectious, and neoplastic diseases. Adult-onset Still’s disease (AOSD), systemic lupus erythematosus (SLE), anti-neutrophil cytoplasm associated-vasculitis (AAV), periodic febrile syndromes such as cryopyrin-associated and tumor-necrosis factor-associated periodic syndromes (CAPS; TRAPS), cryoglobulinemia, Sweet’s disease, and lymphoma are the main alternative diagnosis [2].

The pathogenesis of the SchS is still debated but is generally accepted that SchS patients present a hyperactivation of the inflammasome. A recent study showed augmented neutrophils interleukin-1 receptor antagonist and interleukin-1 beta (IL-1 β) mRNA expression during active disease and a hyperresponsivity to LPS stimulation in SchS patients’ peripheral blood mononuclear cells, producing far more IL-1β and interleukin-6 (IL-6) than normal controls. This is consistent with the previous findings of augmented IL-1β and IL-6 serum levels in SchS patients, with higher levels correlating with more active disease [2, 4].

A great variety of treatments have been proposed for SchS, encompassing colchicine, dapsone, steroids, nonsteroidal anti-inflammatory drugs (NSAIDs), thalidomide, TNF-blocking agents (anti-TNF), antihistamines, intravenous immunoglobulins, plasma exchange, methotrexate, azathioprine, pefloxacin, cyclophosphamide, tocilizumab, rituximab, and others, including traditional Chinese medicine [5, 6].

Coherently with the pathogenetic hypothesis, IL-1 blockade is currently regarded as the most effective therapy in SchS: both anakinra (100 mg/day subcutaneously) and canakinumab (150 mg/month subcutaneously) are used with encouraging results, complete remission rate ranging from 83 to 100% with partial remission for most of the remaining cases. IL-1 blockade reduces inflammatory markers, and it is particularly effective on inflammatory, articular, and cutaneous manifestations, but seems not to alter paraprotein concentration in serum, and its efficacy in preventing lymphoproliferative disorder progression is uncertain [2, 5, 7–9].

## Case report

A 21-year-old woman presented to our outpatient clinic with a 2-year history of recurrent episodes of non-pruritic urticaria and cutaneous angioedema, swelling at proximal interphalangeal joints and wrists, diffuse arthralgias and fever (up to 39 °C), without clear periodicity, nor identified triggering factor, especially in the evening. She had already undergone infectious disease and dermatologic and allergology evaluation and was previously treated with corticosteroids, antihistamines, cyclosporine (100 mg/day), and omalizumab without benefit. She had no family history of fever, rheumatic, dermatologic, or autoimmune diseases. When she came to our attention she was in partial remission with 37.5 mg of prednisone daily. She was admitted to our ward, and extensive laboratory testing was performed, revealing elevated CRP (59 mg/L, normal value 0–5 mg/L) and ESR (74 mm/h, 0–20 mm/h) microcytic anemia (hemoglobin 9.5 g/L, mean cellular value 82.0 fL) and neutrophilic leukocytosis (white blood cells 22. 9 10^9^/L, neutrophils 21.2 10^9^/L). IgM levels were slightly elevated (2.75 g/L, normal value 0.4–2.3 g/L), while IgA, IgE, and IgG levels were normal. Serum protein electrophoresis revealed an inflammatory pattern, without any monoclonal gammopathy, and urinary Bence-Jones protein was negative. Antinuclear antibodies were positive at 1:80, with a homogenous pattern. Other autoimmunity and oncologic and hematologic screenings, infectious diseases, and bone turnover assessments did not show alterations and are summarized in Table 1. Chest X-ray and abdomen US were performed, without evidence of any pathological finding, especially enlarged spleen or bone lesions. PET-CT showed diffuse homogenous bone marrow uptake and slight splenic uptake. A cutaneous biopsy was also performed, revealing leukocytoclastic neutrophilic infiltration with minimal vasculitic aspects and a minimum amount of dermal edema. No pathological findings were found during cardiologic, ophthalmologist, and otolaryngologist evaluations.

**Table 1:** Diagnostic tests performed

Radio immunosorbent test for Immunoglobulin E, angiotensin-converting enzyme, tryptase, C1 inhibitor, complement component 3 and 4, transaminases, γ-glutamyl-transferase, creatine kinase, lactate dehydrogenase, rheumatoid factor, anti-citrullinated protein antibodies, anti- extractable nuclear antigens antibodies, anti-double strand DNA antibodies, cryoglobulins, beta-2 microglobulin, BCR/ABL and Jak2 mutation, infectious disease screening, alkaline phosphatase, and C terminal telopeptide	

The clinical presentation and the histological findings were highly suggestive for SchS, even if the patients lacked monoclonal gammopathy (confirmed with sensitive immunofixation); thus, we decided to try IL-1 blockade therapy.

We started treatment with anakinra 100 mg subcutaneous daily with total remission of urticaria, fever, and arthralgias in 48 h; thus, we decided to confirm SchS diagnosis. Prednisone was tapered and discontinued in 2 months. After 5 months from the beginning of the treatment with anakinra, the patient developed mild neutropenia (N 1600/mmc) and anakinra was switched to 100 mg every other day without relapses, and at the 8 months of treatment, anakinra was further reduced to 3 days a week. After 15 months the patient reported small urticaria lesions on the days off treatment and she switched back to 1 injection any other day with the resolution of the lesions. At the last visit, after 24 months of follow-up, the patient remains asymptomatic. The laboratory tests are normal and without the development of monoclonal gammopathy or remarkable changes in IgM concentrations. Relevant testing trends in time are summarized in Table 2.

**Table 2:** Laboratory findings at baseline and after 5, 12, and 24 months of administration of anakinra

	M0	M5	M12	M24
Therapy	PN 37.5 mg daily	Anakinra 100 mg daily	Anakinra 100 mg every 2 days	Anakinra 100 mg every 2 days
CRP (mg/L)	59	2.3	0.6	0.3
IgM (g/L)	2.75	3.02	3.2	2.88
WBC (*10^9^/L)	22.900	4450	6800	7100
N (*10^9^/L)	21.200	1600	3300	3930
Hb (mg/dl)	9.5	11.6	13.8	13.6

*M* month, *PN* prednisone, *CRP* C-reactive protein, *IgM* immunoglobulin M, *WBC* white blood cells, *N* neutrophils, *Hb* hemoglobin

## Search strategy

We performed a review of the literature available on MEDLINE/Pubmed and Researchgate databases, searched in August 2020, using the following query boxes: ((Schnitzler’s syndrome)AND((without)OR(absence)OR(delayed))AND(monoclonal)AND(gammopathy)); ((Schnitzler’s syndrome)AND((without)OR(absence)OR(delayed))AND(paraprotein)). We analyzed the retrieved articles, focusing on case reports of Schnitzler syndrome without monoclonal gammopathy, deleting double reports, and checking their references for further cases. We summarized the relevant features of retrieved cases in Table 3.

**Table 3:** Literature review of reported cases of Schnitzler’s Syndrome without monoclonal gammopathy and described case comparison

Author, yr	Age (yrs)	Sex	Monoclonal gammopathy (quantity) (FU)	Treatment(s)	IL-1 blockade treatment(s)	Outcome (timing)	AEs
Gladue HS, 2014	58	M	IgM k (20 months)	CS, MTX,ETN, IFX	Anakinra Canakinumab	Initial response	Severe site injection reaction (anakinra) Death (ventricular tachycardia)
	44	F	No (N/A)	Any	Anakinra Canakinumab	Remission	Severe site injection reaction (anakinra)
Husak R, 2000	57	M	Total IgM elevation (6.5 g/L) (24 months)	Antihistamine, CS, trofosfamide, AZA, colchicine, PEX, IFN-α	No		
Varella TCN, 2005	36	M	Total IgM elevation (3.7 g/L) (N/A)	Thalidomide,antihistamine, NSAIDs, IFN-α	No		
Urbanski M, 2016	62	M	No (14 months)	MTX, AZA, dapsone, ADA, colchicine	Anakinra	Remission (24 h)	No
Mulla E, 2015	71	M	Total IgM elevation (4.7 g/L) monoclonal IgM k (48 months)	CS, colchicine	Anakinra	Remission (days)	No
Ahn MJ, 2018	69	M	No (3 months)	ETN, antihistamine, CS, colchicine, CsA, dapsone, MTX	Anakinra	Remission (24 h)	Mild site injection reaction
described case	21	F	Total IgM elevation (2,57 g/L) (24 months)	NSAIDs, CS, CsA, omalizumab	Anakinra	Remission (48 h)	Neutropenia

*yrs.* years, *CsA* cyclosporine, *MTX* methotrexate, *AE* adverse events, *CS* corticosteroids, *NSAIDs* non steroidal anti-inflammatory drugs, *ETN* etanercept, *ADA* adalimumab, *AZA* azathioprine, *PEX* plasma exchange, *IFX* infliximab, *IFN-α* interferon alfa, *FU* follow-up, *N/A* not available

## Discussion

Despite the lacking of a monoclonal gammopathy, which prevented the fulfillment of diagnostic criteria, our case is typical for SchS in many regards: age of onset, histological findings, and response to IL-1 blockade therapy. However, to our knowledge, this is the first report of SchS without monoclonal gammopathy in Italy. We retrieved only 7 further case reports of patients diagnosed with SchS without monoclonal gammopathy [10–15].

The average age at presentation of the disease was 51 years old, mostly males (6/8). Six of them had skin biopsies performed, which were all positive for neutrophilic urticaria.

A wide range of other treatments was reported in these patients: prednisone, NSAIDs and thalidomide, interferon γ, azathioprine, anti-TNF, and methotrexate. Coherently with available data for SchS some of these treatments brought only symptomatic relief, mainly on arthralgias, but failed to achieve remission in any patient. Our patient was treated with omalizumab, which proved to be ineffective in treating inflammatory symptoms of Schs. Indeed, SchS is not an IgE-mediated inflammatory condition, as highlighted also by Nham T et al. [16].

Six patients were treated with IL-1 blocking therapies: anakinra 100 mg daily (*n* = 4) and canakinumab 150 mg every 4 weeks (*n* = 2). One patient was treated with both drugs. All patients achieved remission. Reported IL-1 blockade therapy adverse events were injection site reaction (*n* = 3) and transient neutropenia (*n* = 1). One patient died due to ventricular tachycardia after the beginning of canakinumab treatment, but the patient had pre-existing heart disease.

Follow-up duration was reported for 6 out of 8 patients and varied between 3 and 48 months (average = 18.2 months). Four patients, including the one we described, had slightly elevated total IgM (range 2,57–6.5 g/L; average 4.4 g/L) at diagnosis. Two patients developed IgM k chain monoclonal gammopathy after 20 and 48 months from the initial evaluation (average = 34 months). One of them had previously total IgM elevation and was already in treatment with anakinra, while the other started IL-1 blockade after the finding of monoclonal gammopathy [10, 11]. These findings appear consistent with the reported inefficacy of IL-1 blockade in reducing paraprotein concentration in SchS patients [2, 4].

Lastly, several alternative diagnoses must be ruled out before diagnosing SchS, as this condition shares many clinical features with a number of systemic inflammatory diseases. In our case, the age of onset (21 years old), the lack of fever periodicity, and trigger factors were less typical of CAPS or TRAPS. Absent anti-double strand DNA antibodies, normal complement levels, negative ANA, and not detectable cryoglobulins excluded SLE, AAV, and cryoglobulinemia. The urticarial lesions were also more consistent with SchS than AOSD or Sweet’s syndrome. Finally, the absence of Bence Jones protein, PET-CT findings, and the favorable outcome ruled out lymphoma.

## Conclusions

Is SchS without monoclonal gammopathy a different entity? Husak et al., back in 2000, suggested that the spectrum of SchS should be broadened to include such entity [14]. Our review of the literature retrieved 8 patients with this diagnosis, which would account for a rough 2–3% of the total known cases, even if SchS is probably underdiagnosed [17].

A very high clinical suspicion and an extensive workout to exclude alternative diagnoses are essential to diagnose SchS without monoclonal gammopathy. In this study we also highlight the resolution of fever and rash in all patients treated with IL-1 blockade, but not in patients on other treatments; therefore, we suggest that a trial with IL-1 blockade could be useful to unveil the diagnosis of SchS in the absence of a detectable monoclonal component, as Gusdorf et al. and by Neel et al. suggested for SchS [2, 7].

## Data Availability

No additional data available.
